# Real-world outcomes of treatment strategy between first-line osimertinib, first/second-generation EGFR-TKIs followed by osimertinib and without osimertinib in advanced EGFR-mutant NSCLC

**DOI:** 10.1016/j.esmorw.2024.100058

**Published:** 2024-07-27

**Authors:** Y. Uehara, Y. Takeyasu, T. Yoshida, A. Tateishi, M. Torasawa, Y. Hosomi, K. Masuda, Y. Shinno, Y. Matsumoto, Y. Okuma, Y. Goto, H. Horinouchi, N. Yamamoto, Y. Ohe

**Affiliations:** 1 Department of Thoracic Oncology, National Cancer Center Hospital, Tokyo, Japan; 2 Department of Thoracic Oncology and Respiratory Medicine, Tokyo Metropolitan Cancer and Infectious Diseases Center, Komagome Hospital, Tokyo, Japan; 3 Department of Precision Cancer Medicine, Center for Innovative Cancer Treatment, Graduate School of Medical and Dental Sciences, Tokyo Medical and Dental University, Tokyo, Japan

**Keywords:** epidermal growth factor receptor (EGFR)-mutation, first/second-generation EGFR-TKI, osimertinib, real-world data

## Abstract

**Background:**

Osimertinib has been the standard of care in epidermal growth factor receptor (EGFR)-mutant non-small-cell lung cancer (NSCLC). We evaluated outcomes between osimertinib and first/second-generation (1G/2G) EGFR-tyrosine kinase inhibitors (TKIs) as first-line (1L), and investigated how T790M status and sequential osimertinib after 1G/2G EGFR-TKI failure affected overall survival (OS).

**Materials and methods:**

We retrospectively evaluated the outcomes of patients with advanced NSCLC harboring exon 19 deletion or L858R mutation who received osimertinib and 1G/2G EGFR-TKIs as 1L treatment from January 2015 to March 2021. In the exploratory analysis, we analyzed the outcomes among three groups: osimertinib as 1L (1L-Osi), 1L 1G/2G EGFR-TKIs followed by osimertinib (2L-Osi), and 1L 1G/2G EGFR-TKIs without osimertinib (No-Osi). Propensity score matching (PSM) and 12-month landmark analysis were used to mitigate selection bias and immortal time bias.

**Results:**

Of 485 patients, 213 and 272 received 1L osimertinib and 1L 1G/2G EGFR-TKIs. All 2L-Osi patients had T790M mutations after 1G/2G EGFR-TKI failure. OS did not differ according to 1L EGFR-TKIs [osimertinib versus 1G/2G EGFR-TKIs; 33.7 versus 41.8 months; hazard ratio (HR) 0.92; 95% confidence interval (CI) 0.65-1.29]. In the 12-month landmark analysis, the median OS was 34.4 months [95% CI 21.3 months-not reached (NR)] in 1L-Osi, 63.8 months (95% CI 46.0 months-NR) in 2L-Osi, and 22.5 months (95% CI 19.0-35.3 months) in No-Osi. After PSM, similar results were observed.

**Conclusions:**

There was no significant difference in OS between osimertinib and 1G/2G EGFR-TKIs as 1L treatment in patients with EGFR-mutant NSCLC. However, 2L osimertinib following 1L 1G/2G EGFR-TKIs in patients who would acquire T790M mutation has been linked to a better prognosis compared to 1L osimertinib.

## Highlights

• We compared the real-world data outcomes between 1L-Osi and 1L 1G/2G TKIs.
• In the exploratory analysis, we evaluated outcomes between 1L-Osi, and 1L 1G/2G TKIs with and without Osi.
• OS did not differ between 1L-Osi and 1L 1G/2G TKIs.
• Sequential 1L 1G/2G TKIs and 2L-Osi might show better OS than 1L-Osi.
• Further work focused on the prediction of EGFR-TKI resistance is needed.

## Introduction

Epidermal growth factor receptor tyrosine kinase inhibitors (EGFR-TKIs) have remarkably improved survival outcomes in patients with classical EGFR mutations [L858R or exon 19 deletions (Ex19del)]. First-generation (1G) EGFR-TKIs (gefitinib and erlotinib) reversibly bind to EGFR and inhibit the binding of ATP to its tyrosine kinase domain, while second-generation (2G) EGFR-TKIs (afatinib and dacomitinib) covalently bind to and irreversibly block enzymatically active ErbB receptor family members. The third-generation EGFR-TKI, osimertinib, selectively and irreversibly targets sensitizing EGFR mutations as well as T790M, which is the most common gatekeeper resistance mutation after 1G/2G EGFR-TKI treatment, sparing the wild-type EGFR-tyrosine kinase.^1^^,^^2^ In the FLAURA trial, first-line (1L) osimertinib prolonged progression-free survival (PFS) and overall survival (OS) compared with 1G EGFR-TKIs in patients with EGFR-mutant non-small-cell lung cancer (NSCLC).^3^ Osimertinib has been the standard of care for patients with treatment-naive EGFR-mutant NSCLC.

Second-line (2L) osimertinib also showed superior survival compared with platinum chemotherapy in patients who acquired T790M mutation after 1G/2G EGFR-TKI failure.^4^ In general, T790M mutation occurs in ∼30%-50% of the patients treated with 1G/2G EGFR-TKIs.^4^ Whereas 1L 1G/2G EGFR-TKIs and sequential osimertinib in T790M-positive cases were reported to demonstrate favorable survival outcomes, these outcomes were not compared with those of 1L osimertinib control group.^5^, ^6^, ^7^, ^8^, ^9^, ^10^, ^11^, ^12^, ^13^, ^14^, ^15^ Although the analysis of the genomic and transcriptomic data at baseline may be able to predict the emergence of T790M mutation, predicting the occurrence of T790M mutation before the initiation of 1G/2G EGFR-TKI treatment is extremely difficult.^16^

In this study, we evaluated the real-world clinical outcomes between osimertinib and 1G/2G EGFR-TKIs as 1L treatment. In the exploratory analysis, we investigated how T790M status and sequential osimertinib after 1G/2G EGFR-TKI failure affected OS in order to determine the optimal overall EGFR-TKI treatment strategy.

## Materials and methods

### Study population

We retrospectively reviewed data of advanced or recurrent NSCLC patients treated with 1L EGFR-TKI monotherapy at a single center in Japan between 1 April 1 2015 and 31 March 2021. The inclusion criteria include the following: (i) advanced or recurrent NSCLC with EGFR classical mutations (Ex19del or L858R); (ii) patients who received EGFR-TKIs as 1L treatment as a single agent (gefitinib, erlotinib, afatinib, dacomitinib, or osimertinib).

The patient’s electronic medical records were retrospectively reviewed by investigators to collect data on patient characteristics including age, sex, smoking history, Eastern Cooperative Oncology Group (ECOG) performance status (PS), histological subtypes (adenocarcinoma or others), EGFR mutation status (Ex19del or L858R), disease stage (stage III/IV or recurrence), presence of brain metastasis and liver metastasis, prior brain radiotherapy and surgery, and survival outcome. These data are collected at the point of care. The follow-up period started on the first day of treatment, and the cut-off date was 30 November 2021. The study protocol was approved by the ethics committee of the hospital. This study was reported using the ESMO Guidance for Reporting Oncology Real-World Evidence guidelines for real-world data reporting.^17^

### Study outcome evaluation

The primary endpoint of this study was OS. OS was defined as the interval between the start of 1L EGFR-TKI treatment and the date of death. Patients who were alive at the last follow-up were handled as censored. In general, computed tomography examinations were carried out every 6-8 weeks or according to the attending physician’s judgment. The tumor response was evaluated using RECIST version 1.1. Objective response rate (ORR) was defined as the proportion of patients with at least one confirmed complete response (CR) or partial response (PR) after treatment. Disease control rate was defined as the proportion of patients with CR, PR, or stable disease. The progression of the disease was defined by clinical teams using clinical and radiological assessment. PFS was measured from the time of treatment initiation to clinical or radiographic progression or death from any cause, whichever occurred first. Median follow-up was calculated using the reverse Kaplan–Meier method.^18^

### Propensity score matching

Propensity score matching (PSM) was carried out to minimize the potential selection bias of the retrospective study. The propensity score was generated using a logistic regression model, with the selection of covariates for PSM model informed by prior research, including subgroup covariates of interest from the FLAURA trial.^3^^,^^19^, ^20^, ^21^ Age, sex, smoking history, ECOG-PS, histological subtypes, EGFR mutation status, disease stage, presence of brain metastasis and liver metastasis, and prior brain radiotherapy and surgery were included in PSM. Before PSM was carried out, all patients’ data were anonymized and outcome data were removed. Patients who received 1L osimertinib matched one-to-one with those who received 1L 1G/2G EGFR-TKIs using the nearest neighbor matching algorithm, with a caliper width of 0.2 of the standard deviation of the logit of the propensity score.

### Statistical analysis

Patient characteristics were summarized descriptively using chi-square tests for categorical variables and Mann–Whitney *U* test for continuous variables. No missing values were observed for any of the study variables. Survival analysis was carried out using the Kaplan–Meier method with log-rank *P* values, hazard ratio (HR), and 95% confidence interval (CI) to compare the clinical outcomes between osimertinib and 1G/2G EGFR-TKIs as 1L treatment. Kaplan–Meier analysis was conducted to estimate OS before and after PSM. In the exploratory analysis, we evaluated the difference in OS between osimertinib as 1L (1L-Osi), sequential 2L osimertinib after 1G/2G EGFR-TKIs failure (2L-Osi), and 1L 1G/2G EGFR-TKIs without osimertinib (No-Osi), using 12-month landmark analysis to mitigate immortal time bias. Thus, patients dying within 12 months of the 1L therapy initiation were excluded from the Kaplan–Meier analysis.^22^ As part of the sensitivity analysis, we conducted a landmark analysis, using time points at 6, 18, and 24 months. The follow-up period began at the landmark time points (6, 12, 18, and 24 months). Additionally, to further address immortal time bias, we compared the 2L-Osi group with patients who received 1L osimertinib and any subsequent 2L treatment. We also compared patients who received 1L osimertinib followed by any 2L treatment with those who received 1L 1G/2G EGFR-TKIs followed by any 2L treatment other than 2L osimertinib.

The *P* values were two sided and considered significant if <0.05. All statistical analyses were carried out using R version 4.2.1 (R Project for Statistical Computing; available athttps://www.R-project.org).

## Results

### Patient characteristics

**Figure 1:**
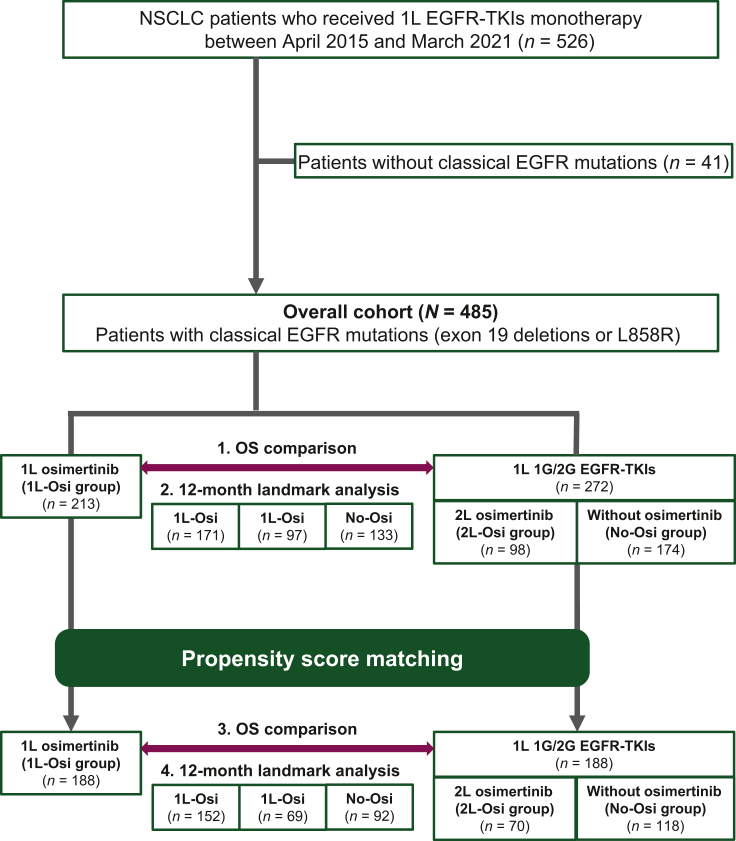
**CONSORT diagram of the study.** 1G, first-generation; 1L, first-line; 2G, second-generation; 2L, second-line; EGFR, epidermal growth factor receptor; NSCLC, non-small-cell lung cancer; OS, overall survival; TKI, tyrosine kinase inhibitor.

A total of 485 patients were included in this study. A total of 213 patients received 1L osimertinib (1L-Osi), and 272 initiated 1L 1G/2G EGFR-TKIs. Of the 272 patients, 98 patients received osimertinib as 2L treatment (2L-Osi), and 174 patients did not receive osimertinib (No-Osi) (Figure 1). All patients in the 2L-Osi group had T790M mutations assessed by tissue or plasma after progression on 1G/2G EGFR-TKIs. Patient characteristics according to types of EGFR-TKI treatments (1L-Osi versus 2L-Osi versus No-Osi) are shown in Table 1. For the whole population, the median age was 68 years (range 27-90 years) (67, 68, and 69 years in 1L-Osi, 2L-Osi, and No-Osi, respectively); females accounted for 66% (67%, 70%, and 63% in 1L-Osi, 2L-Osi, and No-Osi, respectively); almost all (98%) patients had adenocarcinoma histology (97%, 100%, and 97% in 1L-Osi, 2L-Osi, and No-Osi, respectively); EGFR mutation status (Ex19del versus L858R) was 56% versus 44% (59% versus 41%, 63% versus 37%, and 48% versus 52% in 1L-Osi, 2L-Osi, and No-Osi, respectively). Prior and subsequent therapies are shown in Supplementary Table S1, available at https://doi.org/10.1016/j.esmorw.2024.100058. 76%, 15%, and 8% in 2L-Osi received 1L 1G/2G EGFR-TKI treatment (gefitinib, erlotinib, and afatinib, respectively); 69%, 17%, and 14% in No-Osi. The proportions of patients who received their first subsequent therapy were 31%, 52%, and 48% in the 1L-Osi, 2L-Osi, and No-Osi groups, respectively.

**Table 1:** Patient demographic and baseline characteristics

	Total (*n* = 485)	1L osimertinib (1L-Osi) (*n* = 213)	2L osimertinib following 1L 1G/2G EGFR-TKIs (2L-Osi) (*n* = 98)	1G/2G EGFR-TKIs without osimertinib (No-Osi) (*n* = 174)
Median age (range), year	68 (27-90)	67 (28-87)	68 (27-87)	69 (33-90)
Sex, *n* (%)				
Women	322 (66)	144 (67)	69 (70)	109 (63)
Men	163 (34)	69 (32)	29 (30)	65 (37)
Smoking history,^b^*n* (%)				
Never	318 (66)	144 (67)	68 (69)	106 (61)
Former	167 (34)	69 (32)	30 (31)	68 (39)
ECOG-PS, *n* (%)				
0-1	432 (89)	187 (88)	92 (94)	153 (88)
≥2	53 (11)	26 (12)	6 (6)	21 (12)
Histological subtypes, *n* (%)				
Adenocarcinoma	473 (98)	207 (97)	98 (100)	168 (97)
Others^a^	12 (2)	6 (3)	0	6 (3)
EGFR mutation status, *n* (%)				
Exon 19 deletion	270 (56)	125 (59)	62 (63)	83 (48)
Exon 21 L858R	215 (44)	88 (41)	36 (37)	91 (52)
Disease stage, *n* (%)				
III-IV	302 (62)	138 (65)	66 (67)	98 (56)
Recurrence	183 (38)	75 (35)	32 (33)	76 (44)
Brain metastasis, *n* (%)				
Yes	123 (25)	65 (31)	19 (19)	39 (22)
No	362 (75)	148 (69)	79 (81)	135 (78)
Liver metastasis, *n* (%)				
Yes	56 (12)	31 (15)	10 (10)	15 (9)
No	429 (88)	182 (85)	88 (90)	159 (91)
Prior brain radiotherapy, *n* (%)				
Yes	55 (11)	20 (9)	3 (3)	32 (18)
No	430 (89)	193 (91)	95 (97)	142 (82)
Prior brain surgery, *n* (%)				
Yes	5 (1)	3 (1)	0	2 (1)
No	480 (99)	210 (99)	98 (100)	172 (99)

1G, first-generation; 1L, first-line; 2G,second-generation; 2L, second-line; ECOG, Eastern Cooperative Oncology Group; EGFR, epidermal growth factor receptor; PS, performance status; TKI, tyrosine kinase inhibitor.

a Adenosquamous carcinoma (1), carcinosarcoma (2), pleomorphic carcinoma (3), squamous cell carcinoma (4), and not otherwise specified (2).

b All tobacco.

### Clinical outcomes stratified by first-line EGFR-TKI therapy in the overall population

**Figure 2:**
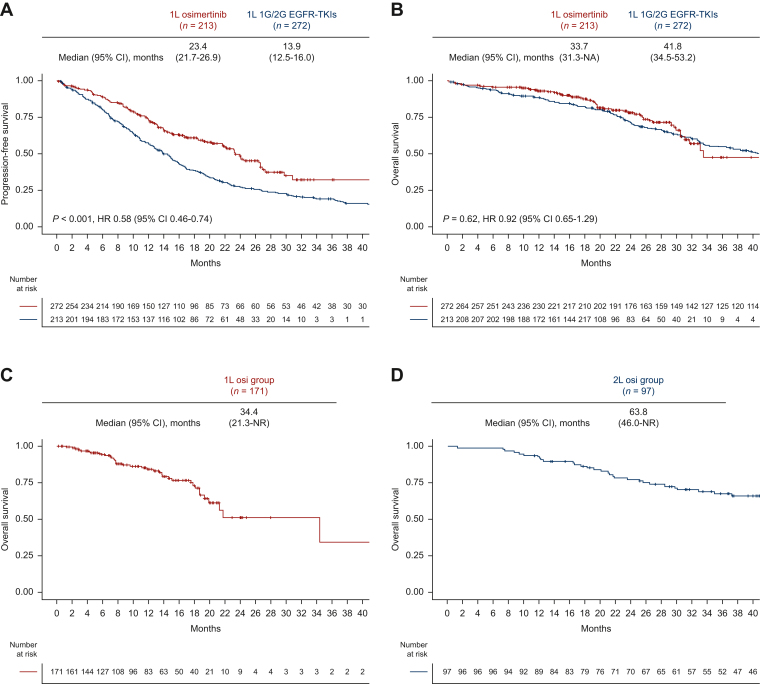

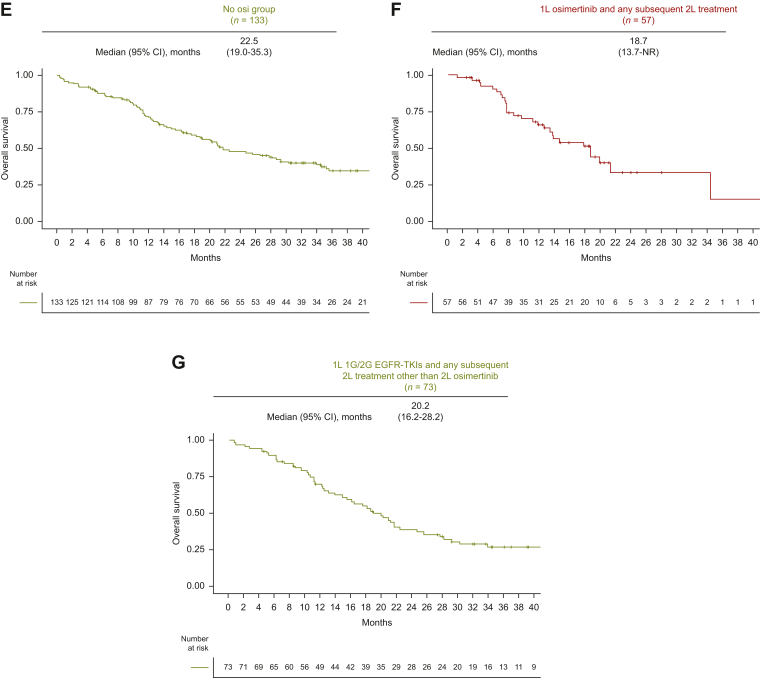
**Kaplan–Meier curves stratified by the strategy of EGFR-TKI therapy before propensity score matching.** (A) PFS of patients treated with 1L osimertinib versus 1L 1G/2G EGFR-TKIs. (B) OS of patients treated with 1L osimertinib versus 1L 1G/2G EGFR-TKIs. 12-month landmark OS analysis of patients treated with (C) 1L osimertinib (1L-Osi), (D) 2L osimertinib following 1G/2G EGFR-TKIs (2L-Osi), (E) 1L 1G/2G EGFR-TKIs without osimertinib (No-Osi), (F) 1L osimertinib and any subsequent 2L treatment, and (G) 1L 1G/2G EGFR-TKIs followed by any 2L treatment other than 2L osimertinib. 1G, first-generation; 1L, first-line; 2G, second-generation; 2L, second-line; CI, confidence interval; EGFR, epidermal growth factor receptor; HR, hazard ratio; NA, not available; NR, not reached; OS, overall survival; PFS, progression-free survival; TKI, tyrosine kinase inhibitor.

The median follow-up was 23.9 months in patients who received 1L osimertinib, and 53.9 months in those who received 1L 1G/2G EGFR-TKIs. The ORR was 70% (95% CI 64% to 76%) in 1L-Osi and 58% (95% CI 52% to 64%) in 1L 1G/2G EGFR-TKIs (Supplementary Table S2, available at https://doi.org/10.1016/j.esmorw.2024.100058). PFS in 1L-Osi was significantly longer than in 1L 1G/2G EGFR-TKIs (median 23.4 versus 13.9 months; HR 0.58; 95% CI 0.46-0.74; *P* < 0.001, Figure 2A). In contrast, OS did not differ according to 1L EGFR-TKIs (osimertinib versus 1G/2G EGFR-TKIs; median 33.7 versus 41.8 months; HR 0.92; 95% CI 0.65-1.29; *P* = 0.62; Figure 2B).

Next, as the exploratory analysis, we evaluated OS according to osimertinib use after 1G/2G EGFR-TKI failure. In the 12-month landmark analysis, the median OS was 34.4 months [95% CI 21.3 months-not reached (NR)] in 1L-Osi, 63.8 months (95% CI 46.0 months-NR) in 2L-Osi, and 22.5 months (95% CI 19.0-35.3 months) in No-Osi (Figure 2C-E). In the sensitivity analysis, the median OS was 18.7 months (95% CI 13.7 months-NR) in patients who received 1L osimertinib followed by any 2L treatment, and 20.2 months (95% CI 16.2-28.2 months) in those who received 1L 1G/2G generation EGFR-TKIs followed by any 2L treatment other than 2L osimertinib (Figure 2F-G). Landmark analyses at 6, 12, 18, and 24 months consistently showed similar results (Supplementary Figure S1A-I, available at https://doi.org/10.1016/j.esmorw.2024.100058).

### Progression patterns after EGFR-TKI failure in the overall population

**Figure 3:**
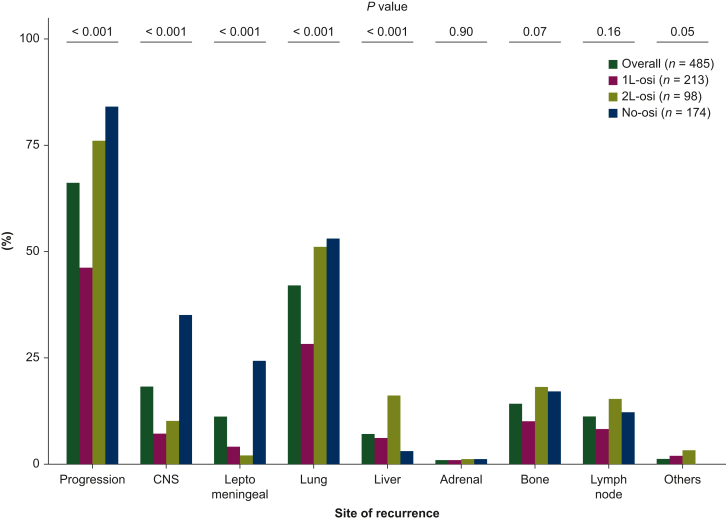
**Progression patterns after EGFR-TKI failure in the overall population.** The percentage of patients with recurrence is shown. Others, peritoneum (*n* = 4), skin (*n* = 1), muscle (*n* = 1), and pancreas (*n* = 1). *P* values were calculated using the chi-square test. 1L, first-line; 2L, second-line; CNS, central nervous system; EGFR, epidermal growth factor receptor; TKI, tyrosine kinase inhibitor.

During the study period, 319 patients (66%) experienced disease progression. The most common progression site was the lung (42%), followed by bone (14%), central nervous system (CNS) (18%), lymph node (11%), leptomeningeal (11%), and liver (7%) recurrence (Figure 3). There were significant differences in the frequencies of CNS, leptomeningeal, lung, and liver metastases progression among the three groups (1L-Osi versus 2L-Osi versus No-Osi) (*P* < 0.001). The CNS progression, including leptomeningeal metastases, was more frequently observed in No-Osi (35%) than in 1L-Osi (7%) and 2L-Osi (10%). The lung progression was less likely in 1L-Osi (28%) than in 2L-Osi (51%) and No-Osi (53%). The liver progression was more likely in 2L-Osi (16%) than in 1L-Osi (6%) and No-Osi (3%).

### Survival outcomes stratified by first-line EGFR-TKI therapy after propensity score matching

In order to compare clinical outcomes between 1L osimertinib and 1L 1G/2G EGFR-TKIs, we carried out PSM to reduce the potential selection bias of the retrospective study. After PSM, 188 patients received 1L osimertinib (1L-Osi), and 188 patients received 1L 1G/2G EGFR-TKIs (Supplementary Table S3, available at https://doi.org/10.1016/j.esmorw.2024.100058). Of the 188 patients receiving 1L 1G/2G EGFR-TKIs, 70 patients received osimertinib following 1G/2G EGFR-TKIs failure (2L-Osi), and 118 patients did not receive osimertinib (No-Osi) (Figure 1). The median follow-up duration in patients who received 1L osimertinib was 20.5 months and in those who received 1L 1G/2G EGFR-TKIs was 33.5 months. Baseline characteristics of the patients were similar between the two groups (*P* > 0.05). In addition, the assessment of the standardized mean differences generated a value <0.1 in all variables (Supplementary Table S3, available at https://doi.org/10.1016/j.esmorw.2024.100058), suggesting that the imbalanced distribution of characteristics was minimized with the matching procedure.^23^

**Figure 4:**
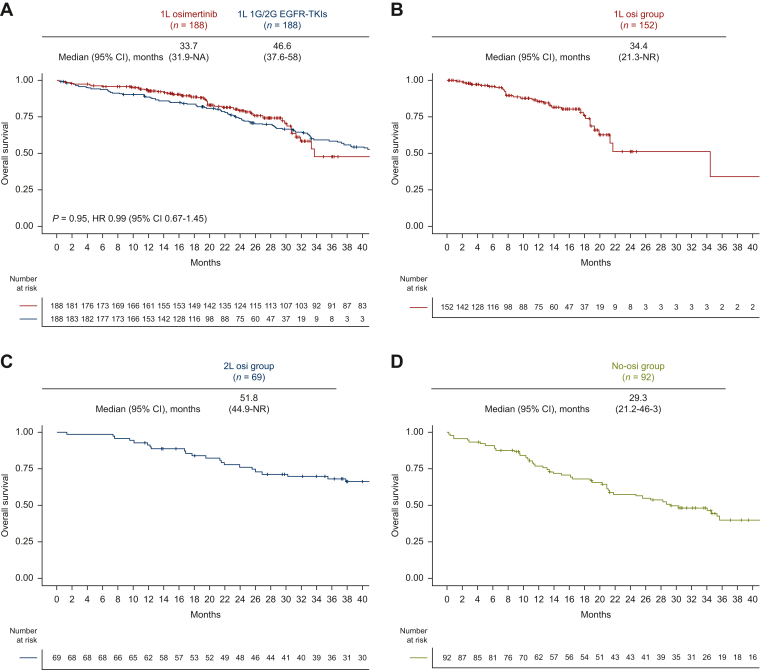

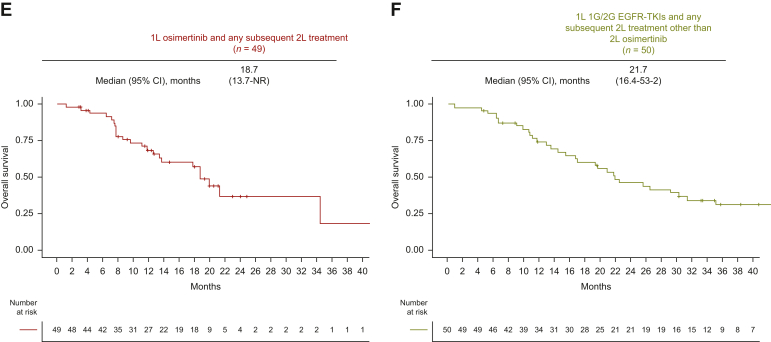
**Kaplan–Meier curves stratified by the strategy of EGFR-TKI therapy after propensity score matching.** (A) OS of patients treated with 1L osimertinib versus 1L 1G/2G EGFR-TKIs after PSM. 12-month landmark OS analysis of patients treated with (B) 1L osimertinib (1L-Osi), (C) 2L osimertinib following 1G/2G EGFR-TKIs (2L-Osi), (D) 1L 1G/2G EGFR-TKIs without osimertinib (No-Osi), (E) 1L osimertinib and any subsequent 2L treatment, and (F) 1L 1G/2G EGFR-TKIs followed by any 2L treatment other than 2L osimertinib. 1G, first-generation; 1L, first-line; 2G, second-generation; 2L, second-line; CI, confidence interval; EGFR, epidermal growth factor receptor; HR, hazard ratio; NA, not available; NR, not reached; OS, overall survival; PSM, propensity score matching; TKI, tyrosine kinase inhibitor.

After PSM, OS also did not differ according to 1L EGFR-TKIs (osimertinib versus 1G/2G EGFR-TKIs; median 46.6 versus 33.7 months; HR 0.95; 95% CI 0.67-1.45; *P* = 0.95; Figure 4A). OS according to subgroups was shown in Supplementary Figure S2, available at https://doi.org/10.1016/j.esmorw.2024.100058. In the exploratory analysis, we evaluated OS according to osimertinib use after 1G/2G EGFR-TKI failure. In the 12-month landmark analysis, the median OS was 34.4 months (95% CI 21.3 months-NR) in 1L-Osi, 51.8 months (95% CI 44.9 months-NR) in 2L-Osi, and 29.3 months (95% CI 21.2 -46.3 months) in No-Osi (Figure 4B-D). In the sensitivity analysis, the median OS was 18.7 months (95% CI 13.7 months-NR) in patients who received 1L osimertinib followed by any 2L treatment and 21.7 months (95% CI 16.4-53.2 months) in those who received 1L 1G/2G generation EGFR-TKIs followed by any 2L treatment other than 2L osimertinib (Figure 4E-F). Landmark analyses at 6, 12, 18, and 24 months consistently showed similar results (Supplementary Figure S3A-I, available at https://doi.org/10.1016/j.esmorw.2024.100058).

## Discussion

Our real-world data demonstrated no difference in OS according to 1L EGFR-TKIs (osimertinib versus 1G/2G EGFR-TKIs) in patients with EGFR-mutant NSCLC, which was consistent with the results of the Asian subset of the FLAURA trial.^3^ In the exploratory analysis, we evaluated OS according to the osimertinib treatment line (1L-Osi, 2L-Osi, and No-Osi group). OS in patients who received sequential 1G/2G EGFR-TKIs and osimertinib was longer than that in those who received 1L osimertinib, whereas OS in patients who received 1L osimertinib was longer than that in those who received 1L 1G/2G EGFR-TKIs without osimertinib after 1G/2G EGFR-TKI failure.

Previous clinical studies have suggested that sequential 1G/2G EGFR-TKIs and osimertinib could be a promising therapeutic approach in patients with EGFR-mutant NSCLC.^5^, ^6^, ^7^, ^8^, ^9^, ^10^, ^11^, ^12^, ^13^, ^14^, ^15^^,^^24^^,^^25^ The phase II APPLE trial demonstrated prospectively that this sequential strategy, applied at the time of progression—whether due to molecular progression (indicated by the detection of the plasma T790M mutation) regardless of radiologic status or solely at radiologic progression—offered a survival benefit comparable to that of 1L osimertinib treatment.^24^^,^^25^ GioTag study retrospectively examined the clinical outcomes of 1L afatinib and sequential osimertinib after the occurrence of T790M mutation.^6^^,^^10^ After a median follow-up of 4.7 years, the median OS was not reached, and the 3-year OS rate was ∼90%. In addition, a *post hoc* study in the randomized LUX-Lung 3, 6, and 7 trials examined 1L afatinib and sequential osimertinib after the occurrence of T790M mutation. After a median follow-up of 4.7 years, the median OS in patients treated with sequential afatinib and osimertinib has not yet been reached, and the 3-year OS rate was ∼95%.^15^ However, in the APPLE trial’s sequential approach arm, some patients switched to osimertinib solely at radiologic progression, regardless of T790M mutation status.^24^^,^^25^ Meanwhile, other studies focused on the patients who acquired T790M mutation after afatinib failure and had no comparator arm with 1L osimertinib.^6^^,^^10^^,^^15^ Indeed, our study showed that sequential 1G/2G EGFR-TKIs and osimertinib showed favorable outcomes compared with 1L osimertinib in patients who acquired T790M mutation after 1G/2G EGFR-TKI failure. These findings suggested that the emergence of T790M mutation at the resistance of 1G/2G EGFR-TKI failure affected total OS after initial EGFR-TKI treatment.

T790M mutation develops in ∼50% of the patients treated with1G/2G EGFR-TKIs.^4^ In a real-world setting, 25%-39% of the patients were reported to receive 2L osimertinib after 1L 1G/2G EGFR-TKIs, which was comparable to our study [36% (98/272) of patients with 1L 1G/2G EGFR-TKIs received 2L osimertinib].^2^ In the FLAURA trial, 47% of patients who started subsequent treatment after 1G EGFR-TKIs received 2L osimertinib.^3^ Recently, Chua et al. identified Ex19del, TP53 wild types, absence of whole-genome doubling, 14q chromosomal amplification, and aging signature as potential predictors for T790M-positive status based on a whole-exome and transcriptome analysis before the onset of 1G/2G EGFR-TKI therapy.^16^ Additionally, the mutational status at the baseline could be a better predictive factor for the efficacy of EGFR-TKIs. Therefore, tumors that would acquire T790M mutation after 1G/2G EGFR-TKI failure could reflect more indolent progression. Indeed, tumors with T790M mutations were reported to be more indolent and less heterogeneous than those without T790M mutations.^26^^,^^27^ Tumors that would acquire T790M mutations after 1G/2G EGFR-TKI failure have a more favorable prognosis than those without T790M mutations regardless of osimertinib treatment. Further work on the development of the prediction on what resistance mechanisms, such as T790M mutation, will emerge after initial EGFR-TKIs is needed to select the appropriate 1L EGFR-TKIs.

Regarding the difference in progression sites of osimertinib, the 2L-Osi group had more liver and lung metastasis progression than the 1L-Osi group. The resistance mechanism of osimertinib has been reported to differ between 1L and 2L osimertinib settings. The proportion of off-target resistance other than EGFR signaling in the 1L setting was higher than that in the 2L setting because 2L osimertinib was used only in tumors with acquired T790M mutation, which demonstrated continued dependence on EGFR signaling.^28^^,^^29^ The distinct resistance mechanisms between 1L and 2L osimertinib could affect distinct progression patterns, such as liver and lung metastases.^28^^,^^30^, ^31^, ^32^ Additionally, patients treated with 1L or 2L osimertinib had a lower incidence of CNS progression including leptomeningeal metastasis. In a preclinical model, osimertinib showed higher CNS penetration than 1G/2G EGFR-TKIs.^33^ In a subgroup analysis of patients with CNS metastases in the FLAURA and APPLE trial, 1L osimertinib showed improved intracranial and survival benefits.^24^^,^^25^^,^^34^ The distinct activity of osimertinib in CNS lesions can lead to the incidence of CNS progression. Further investigation of the clinical outcomes, including progression patterns and resistance mechanism according to the line of therapy and EGFR mutation status, is needed to inform the best practice in the treatment of EGFR-mutant NSCLC.

Several limitations of the present study must be acknowledged. Firstly, there was a bias due to this being a retrospective, single-center study. PSM was carried out to reduce potential selection bias, and most patient background factors were similar between the two groups. Secondly, the follow-up period for patients receiving 1L osimertinib was shorter than that for those receiving 1L 1G/2G EGFR-TKIs, as patients who received 1L osimertinib were enrolled more recently. Although we conducted landmark analyses to address immortal time bias, we cannot completely eliminate the bias from comparing populations from different time periods. Thirdly, we could not include potential predictors of T790M mutation in PSM, such as TP53 wild types and the absence of whole-genome doubling, which we did not measure, although we have included Ex19del in PSM.^16^ Finally, patients who did not receive 2L osimertinib following 1L1G/2G EGFR-TKIs were heterogeneous because some patients could not undergo T790M testing due to the accessibility of tissue.

## Conclusions

In conclusion, there was no difference in OS according to 1L EGFR-TKIs (osimertinib versus 1G/2G EGFR-TKIs) in patients with EGFR-mutant NSCLC, even after PSM. On the other hand, 2L osimertinib following 1L 1G/2G EGFR-TKIs in patients who would acquire T790M mutation after 1G/2G EGFR-TKI failure has been linked to a better prognosis compared to 1L osimertinib. However, it is currently not possible to predict what resistance mechanisms, such as T790M mutation, will emerge at the initiation of first-line EGFR-TKIs. Further work focused on predicting the resistance mechanism of EGFR-TKI is needed to select the optimal EGFR-TKI sequence.
