# Supplementary material for: Real-world outcomes of treatment strategy between first-line osimertinib, first/second-generation EGFR-TKIs followed by osimertinib and without osimertinib in advanced EGFR-mutant NSCLC
Source: ESMO Real World Data Digit Oncol. 2024 Jul 27;5:100058. doi: 10.1016/j.esmorw.2024.100058 (PMC12836644; doi:10.1016/j.esmorw.2024.100058)
Supplement: Supplemental Material [file mmc1.docx]

**Supplementary Table 1 Prior and subsequent therapies.**

| 1L Osimertinib (1L-Osi)  (n = 213) | | 2L Osimertinib following 1L 1G/2G EGFR-TKIs (2L-Osi) (n = 98) | | 1G/2G EGFR-TKIs without osimertinib (No-Osi) (n = 174) | |
| --- | --- | --- | --- | --- | --- |
| First-line therapy, number of patients, n (%) | | | | | |
| Osimertinib | 213 (100) | Gefitinib | 75 (76) | Gefitinib | 120 (69) |
|  |  | Erlotinib | 15 (15) | Erlotinib | 29 (17) |
|  |  | Afatinib | 8 (8) | Afatinib | 25 (14) |
| Second-line therapy | | | | | |
| Received a first subsequent therapy | 65 (31) | Osimertinib | 98 (100) | Received a first subsequent therapy | 83 (48) |
| Cytotoxic chemotherapy | 49 (23) |  |  | Cytotoxic chemotherapy | 53 (30) |
| EGFR TKI | 16 (8) |  |  | EGFR TKI | 27 (16) |
| VEGF inhibitor | 14 (7) |  |  | VEGF inhibitor | 5 (3) |
| Immunotherapy | 5 (2) |  |  | Immunotherapy | 6 (3) |
| Antibody-drug conjugate | 1 (0.5) |  |  | Antibody-drug conjugate | 0 (0) |
| Third-line therapy | | | | | |
| Received a second subsequent therapy | 40 (19) | Received a first subsequent therapy | 51 (52) | Received a second subsequent therapy | 37 (21) |
| Cytotoxic chemotherapy | 21 (10) | Cytotoxic chemotherapy | 50 (51) | Cytotoxic chemotherapy | 20 (11) |
| EGFR TKI | 15 (7) | EGFR TKI | 1 (1) | EGFR TKI | 9 (5) |
| VEGF inhibitor | 9 (4) | VEGF inhibitor | 11 (11) | VEGF inhibitor | 5 (3) |
| Immunotherapy | 1 (0.5) | Immunotherapy | 3 (3) | Immunotherapy | 7 (4) |
| Antibody-drug conjugate | 3 (1) | Antibody-drug conjugate | 0 (0) | Antibody-drug conjugate | 1 (0.5) |

Abbreviations: 1L, first-line; 2L, second-line; EGFR, epidermal growth factor receptor; TKI, tyrosine kinase inhibitor; vascular endothelial growth factor (VEGF); 1G/2G, first-/second-generation.

**Supplementary Table 2 Tumor response stratified by the first-line EGFR-TKI therapy.**

|  | Total  (n = 485) | 1L Osimertinib (1L-Osi)  (n = 213) | 1L 1G/2G EGFR-TKIs  (n = 272) | *P* |
| --- | --- | --- | --- | --- |
| ORR, n (%)  (95% CI) ^a^ | 307 (63)  (59–68) | 150 (70)  (64–76) | 157 (58)  (52–64) | 0.005 |
| DCR, n (%)  (95% CI) ^a^ | 420 (87)  (83–90) | 188 (88)  (83–93) | 232 (85)  (81–90) | 0.41 |
| CR, n (%) | 6 (1) | 4 (2) | 2 (0) | 0.37 |
| PR, n (%) | 301 (62) | 146 (69) | 155 (57) | 0.01 |
| SD, n (%) | 113 (23) | 38 (18) | 75 (28) | 0.02 |
| PD, n (%) | 44 (9) | 15 (7) | 29 (11) | 0.22 |
| NE, n (%) | 21 (4) | 10 (5) | 11 (4) | 0.90 |

^a^ Using exact method based on binomial distribution.

Abbreviations: 1L, first-line; ORR, overall response rate; DCR, disease control rate; CR, complete response; PR, partial response; SD, stable disease; PD, progressive disease; NE, not evaluated; TKI, tyrosine kinase inhibitor; 1G/2G, first-/second-generation.

**Supplementary Table 3 Patient demographic and baseline characteristics before and after propensity score matching**

|  | Before propensity score matching | | | | | After propensity score matching | | | | |
| --- | --- | --- | --- | --- | --- | --- | --- | --- | --- | --- |
|  | Total  (n = 485) | 1L  Osimertinib  (n = 213) | 1L 1G/2G  EGFR-TKIs  (n = 272) | *P* | SMD | Total  (n = 376) | 1L Osimertinib  (n = 188) | 1L 1G/2G  EGFR-TKIs  (n = 188) | *P* | SMD |
| Median age (range), year | 68  (27–90) | 67  (28–87) | 68.5  (27–90) | 0.03 | 0.21 | 67  (27–87) | 67  (28–87) | 67  (27–87) | 0.78 | 0.028 |
| Sex, n (%)  Women  Men | 322 (66)  163 (34) | 144 (67)  69 (32) | 178 (65)  94 (35) | 0.69 | 0.046 | 245 (65)  131 (35) | 126 (66)  62 (33) | 119 (63)  69 (37) | 0.54 | 0.078 |
| Smoking history, n (%)  Never  Former | 318 (66)  167 (34) | 144 (67)  69 (32) | 174 (64)  98 (36) | 0.46 | 0.077 | 248 (66)  128 (34) | 125 (66)  63 (33) | 123 (65)  65 (35) | 0.91 | 0.022 |
| ECOG-PS, n (%)  0–1  ≥2 | 432 (89)  53 (11) | 187 (88)  26 (12) | 245 (90)  27 (10) | 0.51 | 0.073 | 336 (89)  40 (11) | 166 (88)  22 (12) | 170 (90)  18 (10) | 0.62 | 0.069 |
| Histological subtypes, n (%)  Adenocarcinoma  Others^a^ | 473 (98)  12 (2) | 207 (97)  6 (3) | 266 (98)  6 (2) | 0.89 | 0.039 | 365 (97)  11 (3) | 183 (97)  5 (3) | 182 (97)  6 (3) | 1 | 0.032 |
| EGFR mutation status, n (%)  exon 19 deletion  exon 21 L858R | 270 (56)  215 (44) | 125 (59)  88 (41) | 145 (53)  127 (47) | 0.28 | 0.11 | 228 (61)  148 (39) | 114 (61)  74 (39) | 114 (60)  74 (40) | 1 | <0.001 |
| Disease stage, n (%)  III-IV  Recurrence | 302 (62)  183 (38) | 138 (65)  75 (35) | 164 (67)  108 (33) | 0.36 | 0.093 | 242 (64)  134 (36) | 120 (64)  68 (36) | 122 (65)  66 (35) | 0.91 | 0.022 |
| Brain metastasis, n (%)  Yes  No | 123 (25)  362 (75) | 65 (31)  148 (69) | 58 (21)  214 (79) | 0.03 | 0.21 | 84 (22)  292 (78) | 43 (23)  145 (77) | 41 (22)  147 (78) | 0.90 | 0.026 |
| Liver metastasis, n (%)  Yes  No | 56 (12)  429 (88) | 31 (15)  182 (85) | 25 (9)  247 (91) | 0.09 | 0.17 | 46 (12)  330 (88) | 25 (14)  163 (86) | 21 (11)  167 (88) | 0.64 | 0.065 |
| Prior brain radiotherapy, n (%)  Yes  No | 55 (11)  430 (89) | 20 (9)  193 (91) | 35 (13)  237 (87) | 0.29 | 0.11 | 37 (10)  339 (90) | 20 (10)  168 (90) | 17 (9)  171 (91) | 0.73 | 0.054 |
| Prior brain surgery, n (%)  Yes  No | 5 (1)  480 (99) | 3 (1)  210 (99) | 2 (1)  270 (99) | 0.78 | 0.065 | 1 (0)  375 (100) | 1 (1)  187 (99) | 0  188 (100) | 1 | 0.083 |

^a^ adenosquamous carcinoma (1), carcinosarcoma (2), pleomorphic carcinoma (3), squamous cell carcinoma (4), and not otherwise specified (2).

Abbreviations: 1L, first-line; 2L, second-line; ECOG, Eastern Cooperative Oncology Group; EGFR, epidermal growth factor receptor; PS, performance status; SMD, standardized mean differences; TKI, tyrosine kinase inhibitor.

**Supplementary Figure 1** Analysis of overall survival for patients treated with first-line osimertinib (1L-Osi), 2L osimertinib following 1G/2G EGFR-TKIs (2L-Osi), 1L 1G/2G EGFR-TKIs without osimertinib (No-Osi), before propensity score matching, featuring landmark analyses at (**A–C**) 6, (**D–F**) 18, and (**G–I**) 24 months.**
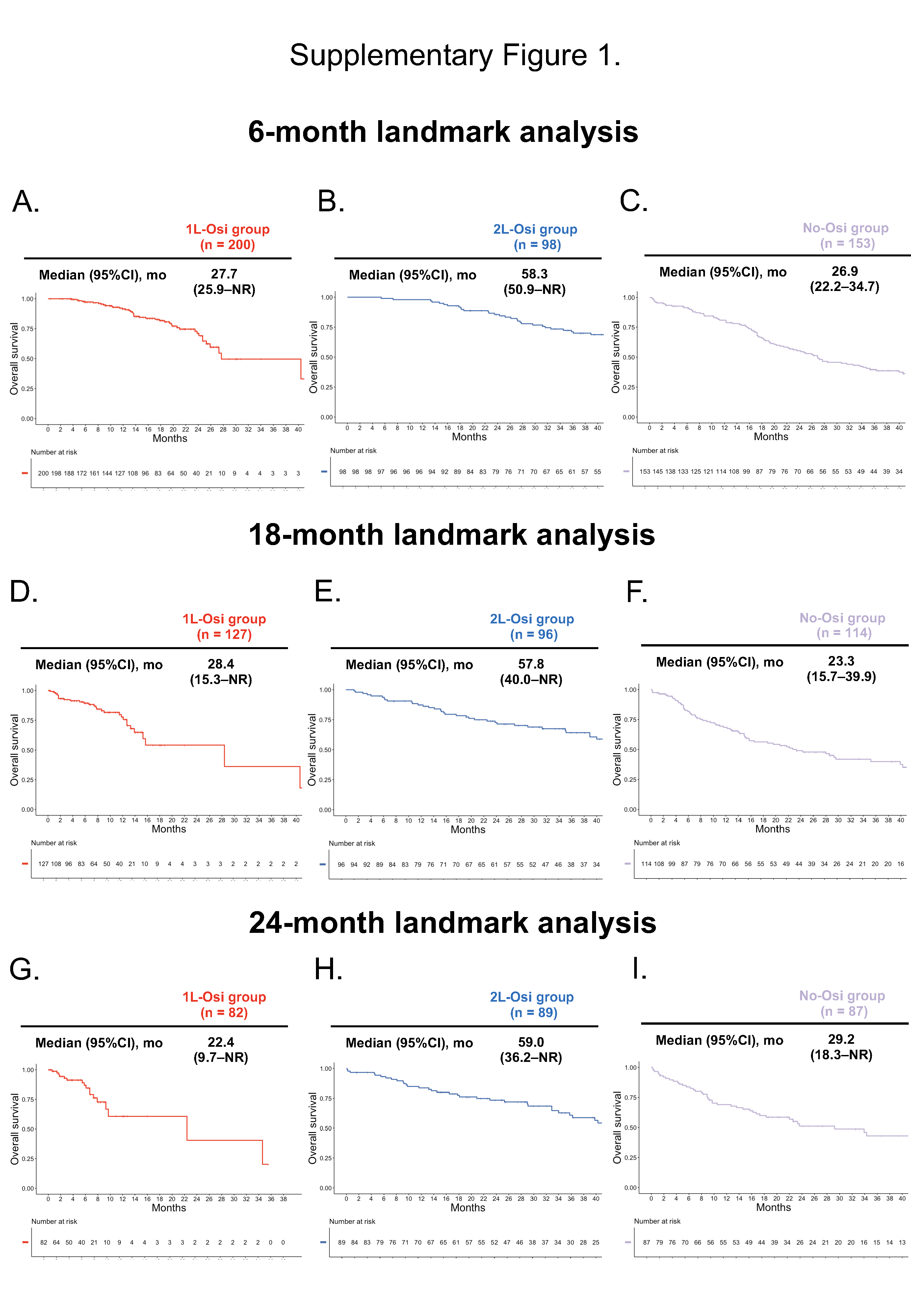
**

**Supplementary Figure 2** Subgroup analyses of overall survival for patients treated with first-line osimertinib versus first-line 1G/2G EGFR-TKIs after propensity score matching.

**
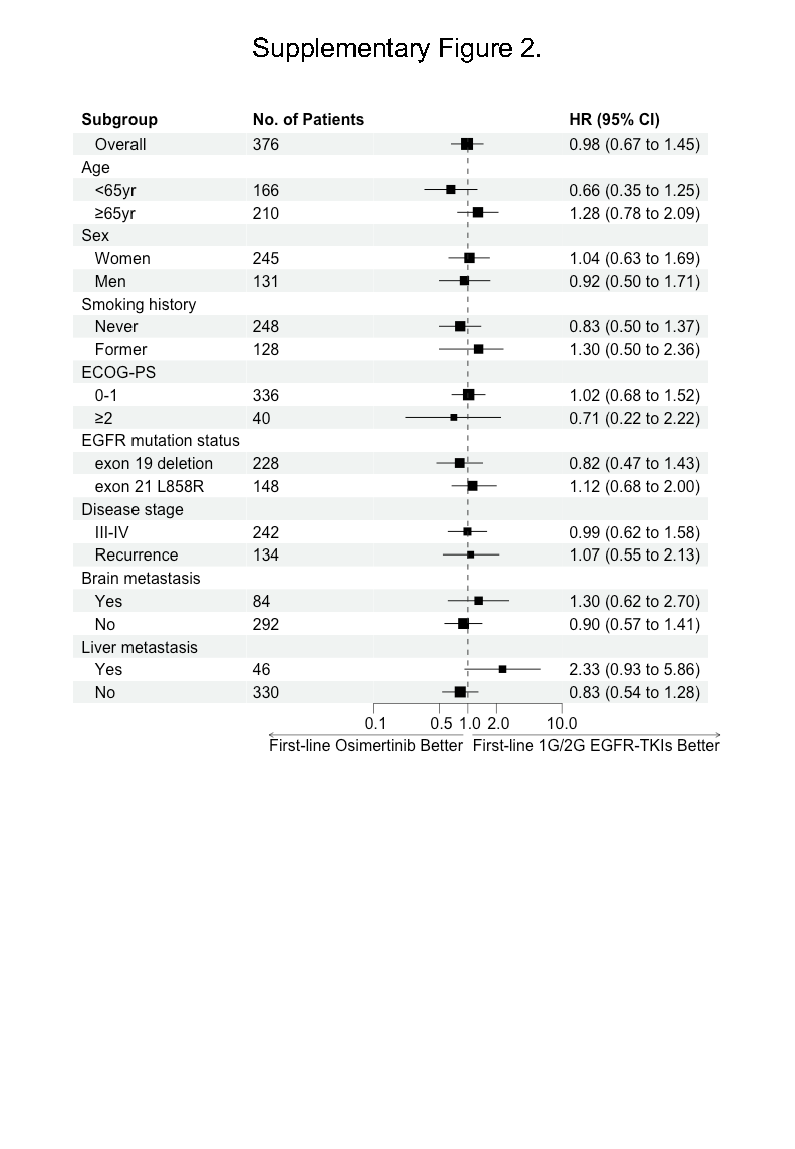
**

**Supplementary Figure 3** Analysis of overall survival for patients treated with first-line osimertinib (1L-Osi), 2L osimertinib following 1G/2G EGFR-TKIs (2L-Osi), 1L 1G/2G EGFR-TKIs without osimertinib (No-Osi), after propensity score matching, featuring landmark analyses at (**A–C**) 6, (**D–F**) 18, and (**G–I**) 24 months.

**
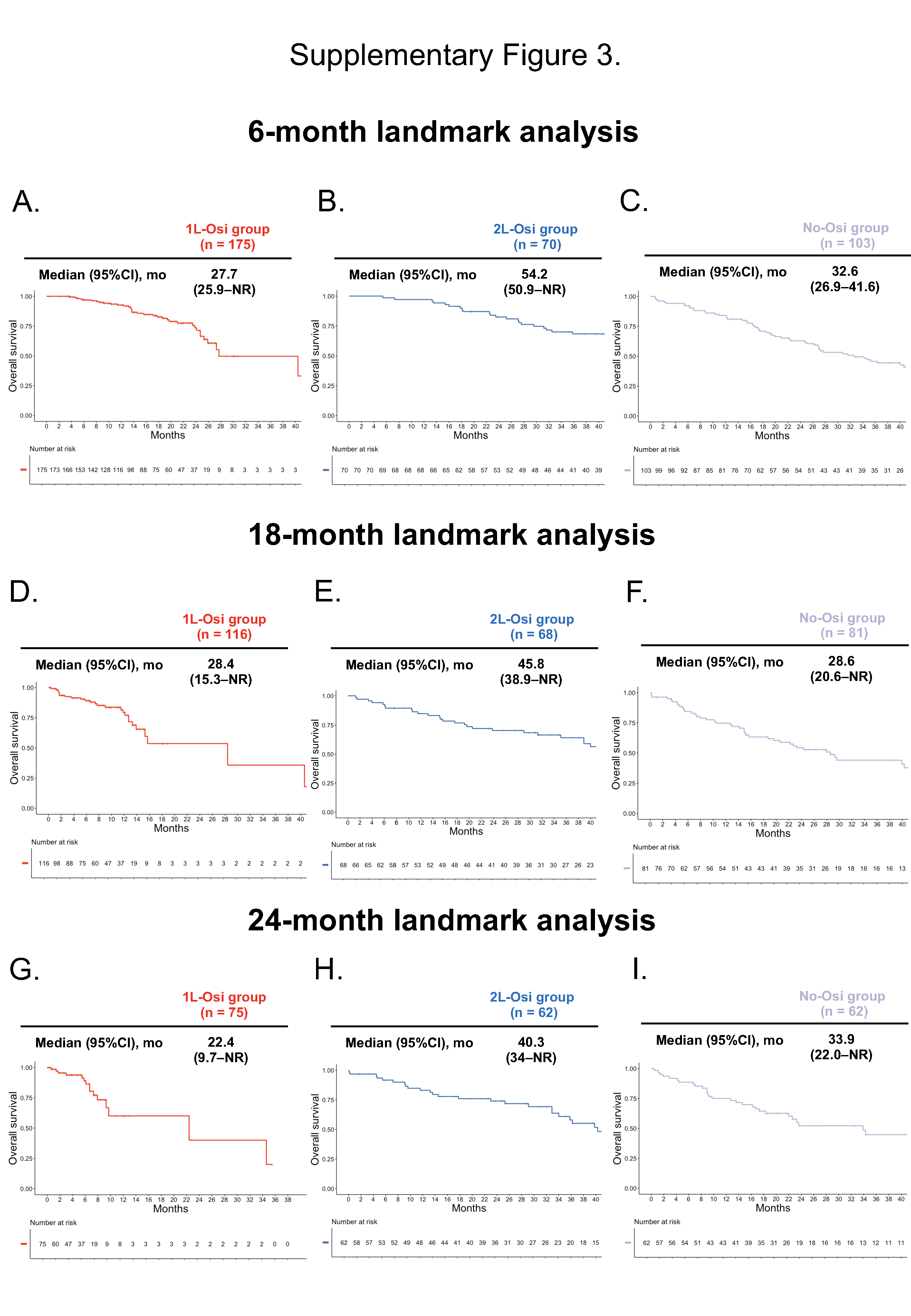
**
